# Different photoperiodic responses in diapause induction can promote the maintenance of genetic diversity via the storage effect in *Daphnia pulex*

**DOI:** 10.1098/rspb.2023.1860

**Published:** 2024-02-14

**Authors:** Yurie Otake, Masato Yamamichi, Yuka Hirata, Haruka Odagiri, Takehito Yoshida

**Affiliations:** ^1^ Department of General Systems Studies, The University of Tokyo, Komaba, Meguro, Tokyo, 153-8902, Japan; ^2^ School of Biological Sciences, The University of Queensland, Brisbane, 4072, Australia; ^3^ Institute of Tropical Medicine, Nagasaki University, Nagasaki, 852-8523, Japan; ^4^ Research Institute for Humanity and Nature, Motoyama, Kamigamo, Kita-ku, Kyoto, 603-8047, Japan

**Keywords:** coexistence, *Daphnia*, diapause induction, diapausing egg, photoperiodic response, storage effect

## Abstract

Understanding mechanisms that promote the maintenance of biodiversity (genetic and species diversity) has been a central topic in evolution and ecology. Previous studies have revealed that diapause can contribute to coexistence of competing genotypes or species in fluctuating environments via the storage effect. However, they tended to focus on differences in reproductive success (e.g. seed yield) and diapause termination (e.g. germination) timing. Here we tested whether different photoperiodic responses in diapause induction can promote coexistence of two parthenogenetic (asexual) genotypes of *Daphnia pulex* in Lake Fukami-ike, Japan. Through laboratory experiments, we confirmed that short day length and low food availability induced the production of diapausing eggs. Furthermore, we found that one genotype tended to produce diapausing eggs in broader environmental conditions than the other. Terminating parthenogenetic reproduction earlier decreases total clonal production, but the early diapausing genotype becomes advantageous by assuring reproduction in ‘short’ years where winter arrival is earlier than usual. Empirically parameterized theoretical analyses suggested that different photoperiodic responses can promote coexistence via the storage effect with fluctuations of the growing season length. Therefore, timing of diapause induction may be as important as diapause termination timing for promoting the maintenance of genetic diversity in fluctuating environments.

## 1. Introduction

How is biodiversity (including genetic and species diversity) maintained despite ecological niche overlap and competition for limited resources? Researchers have investigated this question in evolutionary biology and community ecology for decades [1–3]. One important factor that can promote the maintenance of genetic variation and species coexistence is the production of diapausing stages in fluctuating environments. Many organisms suspend development temporarily in seasonally varying habitats (reviewed in [4,5]). Previous theoretical and empirical studies demonstrated that diapausing stages result in overlapping generations and can promote coexistence of competing genotypes, lineages, or species via the storage effect [6–13]. When reproductive success varies temporally, diapausing stages enable competing genotypes to store products of favourable periods for the purpose of avoiding extinction during unfavourable periods. Without diapausing stages, genotypes with the highest geometric mean growth rate will dominate, and other genotypes will go extinct [6,14,15]. As the ongoing climate change increases extreme weather events, the storage effect with dormancy may become more important in the future [16].

In addition to temporal variation in reproductive success (e.g. seed yield in annual plants), previous studies have shown that variation in the timing of diapause termination, which causes intra-year [17–20] or year-to-year [18,20–22] asynchronous recruitment, can buffer temporal variation in reproductive success and contribute to coexistence of competing genotypes or species via the so-called storage effect, as shown both theoretically [17–19] and empirically [20–22]. Compared to timing of diapause termination, previous studies on the temporal storage effect have not examined the importance of different timing of diapause induction for promoting stable coexistence in fluctuating environments. Researchers have intensively studied adaptive evolution in the optimal timing of diapause induction [23–26], but its potential impact on the maintenance of genetic variation via the storage effect has not attracted so much attention, although timing of diapause induction can be as important as diapause termination.

Here we propose that differences in timing of diapause induction can also contribute to coexistence via the storage effect. Consider a situation where two competing genotypes have different timings of diapause induction and actively reproducing individuals cannot survive after a certain catastrophic event (e.g. the arrival of winter). Earlier production of diapausing offspring enables a genotype to ensure contributions to egg/seed banks even when a growth period is short, whereas later production of diapausing offspring results in longer growth periods for clonal reproduction and larger contributions to egg/seed banks when a growth period is long. Thus, for example, when winter comes earlier than usual in temperate lakes, an active water flea (*Daphnia*) population of a late-diapausing genotype may disappear before they produce diapausing eggs due to low temperatures, while an early-diapausing genotype produces a small number of diapausing eggs. On the other hand, when winter comes later than usual, the late-diapausing genotype produces more clonal offspring and thus will produce more diapausing eggs than the early-diapausing genotype. These kinds of inter-annual fluctuations in the length of the growing season may produce ‘good’ and ‘bad’ years for competing genotypes, enabling stable coexistence via the temporal storage effect.

In this study, we explored whether differences in timing of diapause induction can contribute to the maintenance of intraspecific genetic variation, and whether photoperiodic environments cause differences in timing of diapause induction between genotypes, by using two parthenogenetic (asexual) genotypes of *Daphnia pulex* found in Lake Fukami-ike (Nagano Prefecture, Japan). In lake Fukami-ike, water temperature in winter is usually below 6°C through the water column ([27], A Yagi 1978–2016, unpublished data). Such a low temperature decreases phytoplankton production. Because of the low temperature and resulting food shortage for *Daphnia*, it is likely that *D. pulex* needs to switch to a diapausing state for overwintering. To explore whether different photoperiodic responses in diapause induction contribute to coexistence of two genotypes, we constructed and analysed a mathematical model. Model parameterization took into consideration the results of two experiments using *D. pulex*. First, we examined the effects of photoperiod on the speed of clonal reproduction (via parthenogenetic reproduction) between the two genotypes during the active growing phase. Second, we measured the tendency to produce diapausing eggs under different conditions of food abundance and photoperiod.

## 2. Material and methods

### (a) Experimental materials

Here, we focused on *Daphnia pulex*, one of the keystone species in lake ecosystems [28]. Genus *Daphnia* has often been studied to understand the maintenance of genetic variation [29–32] and species coexistence [33]. Some prior studies demonstrated differences in life-history traits of diapause (timing of diapause induction and termination) between genotypes or species [10,33–38]. Genus *Daphnia* usually propagates by parthenogenetic reproduction but produces diapausing eggs by sexual reproduction in harsh environments, induced by several cues including food abundance, temperature, chemicals released from fish (kairomones) and photoperiod, in addition to overabundance (reviewed in [37]). Photoperiod is an accurate predictor of seasonal changes and thus may be responsible for different timing of diapause induction [37–40]. However, the role of photoperiodic responses in promoting coexistence of competing genotypes, lineages or species has seldom been discussed.

*Daphnia pulex* in Japan is composed of four lineages (JPN1, JPN2, JPN3 and JPN4), and all of them are obligate parthenogens [41]. Furthermore, they produce diapausing eggs by clonal reproduction [41], and thus we can evaluate the effect of the variation in timing of diapause induction on coexistence via the storage effect without considering sexual interactions. Each lineage has various genotypes that are identified with genetic markers in the control region of mitochondrial DNA and NADH dehydrogenase 5 (ND5), and we use ‘clone’ here to refer to individuals of a culture line that are established from a single female in the laboratory. Several prior studies revealed that multiple lineages in Japanese *D. pulex* coexist in a few lakes despite their ecological similarities [41] and they have both intra- and inter-lineage variation in life history and morphological traits [42]. However, to our knowledge, no study has examined variation in their diapausing traits. We studied Lake Fukami-ike (35°19N, 137°49E), one of the lakes in which two lineages of *D. pulex* coexist. The lake is naturally formed and is currently eutrophic, with a maximum depth of 7.8 m and a surface area of 2.2 ha [43]. We collected and analysed the varved sediment core samples from Lake Fukami-ike in a previous study [44]. The previous study found that one genotype of the JPN2 lineage, Jpn2C, established *ca* 2002–2003 and dominated throughout the research period, and then genotypes belonging to the JPN1 lineage, Jpn1A-C2T2 (more abundant) and Jpn1A-C1T2 (scarce), migrated to Lake Fukami-ike around 2013. Since then, these genotypes, belonging to different lineages, coexisted for about three years in the lake. However, it seems that they went extinct around 2016 probably due to an increase in planktivorous invasive fish [45]. Here we focused on Jpn1A-C2T2 and Jpn2C, which are the dominant genotypes in the JPN1 and JPN2 lineages, respectively, and asked whether these genotypes stably coexisted and if so, what mechanisms would promote their longer coexistence.

We isolated *D. pulex* ephippia from the layers of the sediment core samples in which the two distant genotypes, Jpn1A-C2T2 and Jpn2C, coexisted. We then decapsulated the ephippia, isolated diapausing eggs, and exposed the eggs to hatching stimuli, i.e. a long-day photoperiod (16 : 8 h light (L):dark (D)) and 18°C [46]. Hatched individuals were kept in monoclonal cultures in aged tap water under laboratory conditions (23°C, 14 : 10 h L:D, fed 0.63 mg carbon l^−1^ of chemostat-grown green algae, *Scenedesmus*: UTEX1359). These clones were identified genetically based on mitochondrial markers in ND5 with primers ND5 f21 and ND5 r1046 [41] and the control region of mitochondrial DNA with primers IAIT fw and IAIT rv1 [41,47]. Full details of these genetic identification processes are described in our previous study [45]. We used seven clones of each genotype, Jpn1-C2T2 and Jpn2C; thus, we used 14 clones in total. The mother individuals hatched from different diapausing eggs, which were picked from different ephippia, respectively.

### (b) Laboratory experiment 1: clonal reproduction

To evaluate the speed of reproducing active populations in the water column, we conducted experiments between two *D. pulex* genotypes with all possible combinations of seven clones (i.e. a total of 49 combinations) under two photoperiodic conditions: long-day (14 : 10 L : D) and short-day (10 : 14 L : D). The temperature was set to be 18°C according to natural conditions in the spring season in Lake Fukami-ike. We had three replicates for each combination under the two photoperiodic conditions (i.e. 49 × 3 × 2 = 294 in total). Experimental jars were filled with 180 ml aged tap water and four newborn female juveniles of one clone per genotype were added to each jar (i.e. eight individuals were in a jar at the beginning of the experiment). The newborn females were obtained using the following procedure: we transferred clutched females from subculture lines to each experimental photoperiodic condition and the first-generation offspring (generation 1) of each genotype born within a day were transferred to a 200 ml jar that was filled with aged tap water, which was refreshed every 4 days. We fed green algae, *Scenedesmus*, at high food concentrations (0.63 mg C l^−1^) every two days from day 0 (i.e. the first day of the experiment) to day 15. After that, we continued the experiment without food until day 30. On days 15 and 30, we sampled individuals after mixing the contents of the jars. On day 15, 40 ml from each jar were poured into a Petri dish and all animals present were fixed with 99% EtOH in a 1.5 ml microtube. On day 30, all living animals that remained in each experimental jar were fixed with 99% EtOH in a 5 ml microtube. Fixed animals were stored at –30°C.

The change in the frequency of each clone was analysed based on the polymerase chain reaction–restriction fragment length polymorphism (PCR–RFLP) method following H Ohtsuki and J Urabe 2015 (personal communication; they developed the method following [48]). Individuals (8–16) were isolated from each sample and fixed with 95% EtOH, and then they were dried for a day. To extract DNA from these individuals, we added 25 µm Quick Extract DNA Extraction Solution (Lucigen, Wisconsin, US) per individual and subjected them to thermal shock of 60°C for 2 h and 95°C for 20 min. PCR amplification of fragments of ND5 of mitochondrial DNA (775 bp) was performed using ExTaq Hot start Version (Takara) and the primer set comprising DpuND5b and DpuND5n. Each 5 µl reaction consisted of 0.5 µl of extracted DNA, 0.02 µl of TaKaRa Ex Taq HS, 0.4 µl of dNTP, 0.5 µl of 10 × ExTaq buffer and 0.2 µmol l^−1^ of each primer. The thermal cycling conditions were as follows: 94°C for 2 min, followed by 30 cycles of 95°C for 30 s, 48°C for 30 s and 72°C for 1 min, and finished at 72°C for 10 min. We then conducted PCR–RFLP with two restriction enzymes, Hpy 188III and MnII. Individuals' lineages were identified based on the band pattern. Each 10 µl reaction consisted of 5 µl of PCR products, 1.0 µl of CutSmart Buffer (New England Biolabs) and 0.05 µl of each restriction enzyme. The thermal shock consisted of 37°C for 2 h and 65°C for 20 min. Electrophoresis was performed at 50 V for 60 min on a 2% agarose gel. Based on PCR–RFLP, we counted the individual numbers of Jpn1A-C2T2 and Jpn2C.

The results of each combination were evaluated statistically to determine whether Jpn1A-C2T2 or Jpn2C dominated. We used the *t*-test to check whether the genotype frequency changed significantly at the end of the experiment (day 30) from the initial ratio of Jpn1A-C2T2:Jpn2C = 1:1 on day 0. We then calculated the percentage of Jpn2C as the fraction of the number of clone combinations in which the frequency of Jpn2C significantly exceeded that of Jpn1A-C2T2 at day 30. To evaluate the speed of clonal reproduction of each genotype in each photoperiodic condition, we compared the percentage of Jpn2C between the long-day and short-day conditions with two-sided tests of equality of proportions using the ‘prop.test’ function of R [49].

### (c) Laboratory experiment 2: life history measurement

We measured the prevalence of diapause from 14 clones in each photoperiodic condition (long-day or short-day) as the ratio of ephippial females to the total number of reared individuals. We used third-generation offspring (F3) to reduce the potentially confounding maternal and grandmaternal effects [50]. First, clutched adult females (F0 generation) were isolated from subculture lines and reared individually in 200 ml jars under long-day or short-day experimental photoperiodic conditions. When the second-generation offspring (F2) were born, they were reared individually in 200 ml jars under four experimental conditions: (1) long-day and high-food, (2) long-day and low-food, (3) short-day and high-food, and (4) short-day and low-food. We reared F3 newborn females individually in 50 ml plastic tubes under the above-mentioned four experimental conditions until the release of the third clutch. We changed the water and fed them green algae, *Scenedesmus* (low-food (0.063 mg C l^−1^) and high-food (0.63 mg C l^−1^)) every two days. During this experiment, we observed the reared females daily and recorded whether they had parthenogenetic eggs or ephippia, or they released parthenogenetic offspring or ephippia. When parthenogenetic offspring were born, we determined their sex and counted the number of female and male offspring under a stereo microscope (Nikon smz1500). Genotypes belonging to JPN1 can produce male offspring, but males do not contribute to reproduction [41]. Male offspring were also counted but were not used to calculate the intrinsic population growth rate.

In addition to calculating the prevalence of diapause in each clutch of each clone, to understand the mechanism behind the competition experiment, we calculated the intrinsic population growth rate (*r*) based on the data until the third clutch of each clone using the Euler–Lotka equation [51]: $1=\sum_{x=1}^{k}l_{x}m_{x}e^{-rx}$, where *x* is the time length of the experiment in days, *k* is the day of the third clutch for each individual, *l_x_* is the survival probability at day *x*, and *m_x_* is the number of offspring released on day *x*. Because no individuals died during the experiment, we assumed that *l_x_* = 1. As values of *k* and *m_x_* were obtained from the experiment, we could calculate *r* based on the Euler–Lotka equation. To test the effects of genotype (Jpn1A-C2T2 or Jpn2C), photoperiodic conditions, food conditions, and their interactions (explanatory variables) on the prevalence of diapause (a response variable), we analysed the data using a three-way ANOVA. We then compared the prevalence of diapause and intrinsic population growth rate between experimental conditions by multiple comparisons based on Tukey's honestly significant difference (HSD) test followed by Bonferroni correction. In addition, to estimate the effect of the production of diapausing eggs on the competitive ability of each genotype, we tested whether there is a trade-off between the intrinsic population growth rate and prevalence of diapause by the generalized linear model (GLM) with gamma distribution and log-link function. Similarly, a trade-off between the production of parthenogenetic eggs and ephippia was tested by GLM with Poisson distribution and log-link function. In both of these analyses, we tested the effect of prevalence of diapause or the numbers of ephippia from the first to third clutch, genotype, and their interaction (explanatory variables) on intrinsic growth rate or the numbers of parthenogenetic offspring (response variables). All statistical analyses were performed using R version 3.5.2 [49], and *p* < 0.05 was considered significant.

### (d) Theoretical model analysis

**Figure 1.**
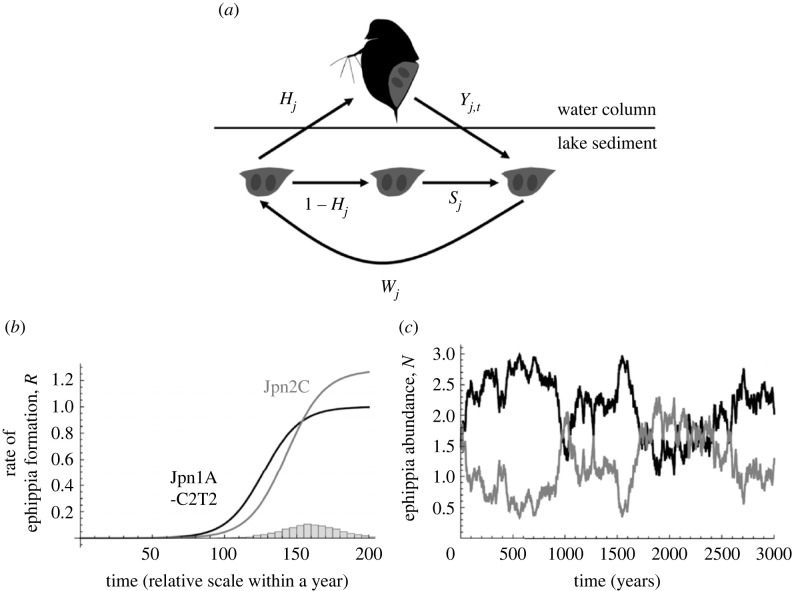
Assumptions of the theoretical analysis (*a,b*) and an exemplifying simulation run of stable coexistence of the two genotypes via the temporal storage effect (*c*). (*a*) The dynamics of diapausing eggs in equation (2.1). (*b*) Lines represent cumulative curves of produced diapausing eggs (the black line is Jpn1A-C2T2 and the grey line is Jpn2C). Note that the values on the horizontal axis represent the relative timing of diapause induction, and thus the absolute values themselves are not significant. Parameter values are *a*_1_ = 1, *a*_2_ = 1.28, *b*_1_ = 0.0783, *b*_2_ = 0.0764, *c*_1_ = 127 and *c*_2_ = 143. The histogram in (*b*) indicates the inter-annual fluctuation in the length of growth season *e_t_* (in this panel, *µ* = 160 and *σ* = 20). Based on the laboratory experiments, we assumed that Jpn1A-C2T2 starts producing diapausing eggs earlier than Jpn2C, and Jpn2C produces more diapausing eggs than Jpn1A-C2T2 when the growth period is long (electronic supplementary material, appendix S1). If the length of the growing season fluctuated among the years as shown by the histogram in (*b*), the two genotypes can stably coexist (*c*). Other parameter values are *w*_1_ = *w*_2_ = 0.9, *H*_1_ = *H*_2_ = 0.1, *s*_1_ = *s*_2_ = 0.9 and *K* = 1.

Using a theoretical model (figure 1*a*), we tested whether the observed difference in timing of diapause induction of the two genotypes, Jpn1A-C2T2 and Jpn2C, can promote coexistence or not. In a difference equation of diapausing egg dynamics of two genotypes [8,33], the number of diapausing eggs of genotype *j* (*j* = 1, 2) at year *t* (*N_j_*_,*t*_) changes as follows:

2.1 $$N_{\;j,t+1}=[\left(1-H_{j})s_{j}+H_{j}Y_{j}(e_{t}\right)]w_{j}N_{\;j,t},$$

where *H_j_* is the hatching rate of diapausing eggs, *s_j_* is the probability of survival of diapausing eggs during summer in the egg bank, *Y_j_*(*e_t_*) is the temporally fluctuating *per capita* production of diapausing eggs (a function of the length of the growing season *e_t_*) and *w_j_* is the probability of survival of diapausing eggs during winter in the egg banks. Assuming the ‘saturating yield’ model [52], *Y_j_*(*e_t_*) can be written as:

2.2 $$Y_{j}(e_{t})=\frac{KR_{j}(e_{t})}{\sum_{k=1}^{2}H_{k}R_{k}(e_{t})N_{k,t}},$$

where *K* is the total production of diapausing eggs after the growth season and *R_j_* is the temporally fluctuating relative production of diapausing eggs of genotype *j*. The subscript *k* in the denominator also represents a genotype. The saturating yield model assumes density-independent production of total diapausing eggs, but there is an equilibrium density and ‘results obtained for the saturating yield model generalize qualitatively to some more realistic models of density-dependent competition’ [8, p. 406]. When the length of the growing season fluctuates, it is likely that both *K* and *R* change temporally. For simplicity, however, here we assumed that *K* is a constant (as like [8]) and *R* changes depending on the length of the growing season. As the relative amount of diapausing eggs produced in the focal year should be an increasing function of the length of the growing season, we here assume that *R_j_* can be represented as a sigmoidal function for cumulative production of diapausing eggs by the end of the growing season, *e_t_*:

2.3 $$R_{j}(e_{t})=\frac{a_{j}}{1+\exp[b_{j}(c_{j}-e_{t})]},$$

where *a_j_* is the maximum production of diapausing eggs by the active individuals of genotype *j* in the water column, *b_j_* is the shape parameter representing the steepness of diapausing egg production, and *c_j_* is the threshold to reach half of the maximum production of diapausing eggs. Thus, an early-diapausing genotype may have a smaller *c* value as it starts producing diapausing eggs earlier than a late-diapausing genotype. However, because of the intrinsic trade-off between production of diapausing eggs and propagation of active individuals (electronic supplementary material, figure S3), the early-diapausing genotype may have a smaller *a* value than the late-diapausing genotype (figure 1*b*). It should be noted, however, that the relative production of diapausing eggs of the early-diapausing genotype can be larger than that of the late-diapausing genotype when the length of the growing season is short (e.g. when *e_t_* < 0.7 in figure 1*b*). We constructed a model for seasonal dynamics within a year assuming season-dependent growth rate and prevalence of diapause, and used parameters estimated from our life-history experiments to obtain the *R* functions (electronic supplementary material, appendix S1, figure S1). Although we estimated the parameters based on the laboratory experiments, these can differ from the actual prevalence of diapause in the wild, especially when diapause induction is affected by other additional factors (e.g. the presence of predators). It will be important to develop a method to handle such parameter uncertainty in future studies. We assumed an inter-annual fluctuation in the length of the growing season, *e_t_*. The timing of winter's onset may vary from year to year in temperate lakes, as seen in the inter-annual variation of lake ice phenology [53]. As *D. pulex* populations cannot grow in the low temperatures of winter [54], this results in inter-annual fluctuations in the length of the growing season. In equation (2.3), the parameter *e_t_* represents the length of the growing season, and we assumed that it randomly fluctuates according to a normal distribution with mean *μ* and variance *σ^2^* (and with the constraint *e_t_* > 0).

When only one genotype exists, the equilibrium abundance of diapausing eggs is $\bar{N_{j}}=Kw_{j}/(1-\gamma_{j}w_{j})$ and the amount of generation overlap is $\gamma_{j}=(1-H_{j})s_{j}$. Coexistence of two genotypes occurs when each genotype can increase when rare, meaning that the geometric mean of the following invasion growth rate is larger than 1 (or the arithmetic mean of log(*N_i_*_,*t* + 1_/*N_i_*_,*t*_) is larger than 0, where the subscript *i* indicates ‘invader’):

2.4 $$\frac{N_{i,t+1}}{N_{i,t}}=\left[(1-H_{i})s_{i}+\frac{KH_{i}R_{i,t}}{H_{j}R_{j}{\bar{N}}_{j}}\right]w_{i}\;\;(i,j=1,2).$$

Based on the laboratory experiments, we assumed that Jpn1A-C2T21 starts to produce diapausing eggs earlier than Jpn2C (*c*_1_ *<* *c*_2_). Meanwhile, Jpn2C can produce more diapausing eggs at the end of the long growing season than Jpn1A-C2T2 (*a*_1_ *<* *a*_2_) because of the higher proportion of active individuals of this genotype at the end of a long growing season. We evaluated the effects of inter-annual growing season fluctuation, diapausing strategies of the two genotypes, and competitive ability in the water column on coexistence of the competing genotypes by numerical simulations of invasion growth rates. We conducted simulations using Wolfram Mathematica 13.3 [55], and evaluated invasibility based on simulations with 10 000 years.

## 3. Results

### (a) Clonal reproduction of the active population and prevalence of diapause

**Figure 2.**
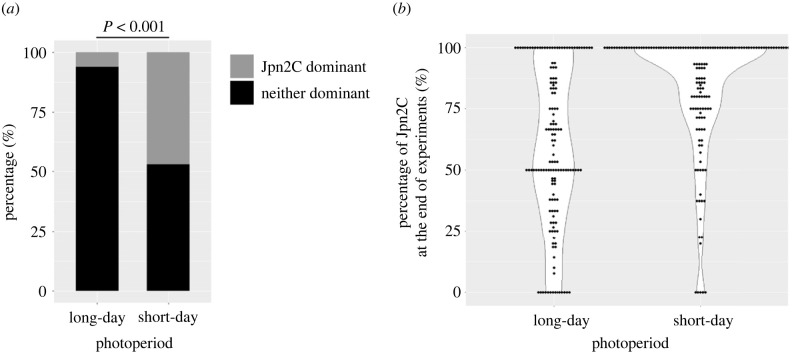
Percentages of matches where Jpn2C dominated (grey) and neither dominated (black) on day 30 under the long-day condition (14 : 10 h light : dark) and short-day condition (10 : 14 h light : dark). (*a*) No match was dominated by Jpn1A-C2T2. Results were detected using the *t*-test. Whether the results were different between long-day and short-day was tested by two-sided tests of equality of proportions. (*b*) The violin plot representing percentages of Jpn2C on the last day of the experiment. Points represent the percentages of Jpn2C in each match. White areas represent the data distributions. The plot was made using ggplot2 package of R [56].

In the clonal reproduction experiments, we found that no match was dominated by Jpn1A-C2T2 and the outcomes were either the dominance of Jpn2C or the domination of neither (figure 2). The fraction of the clone combinations where Jpn2C clones dominated was significantly higher in the short-day condition (46.94%) than in the long-day condition (6.12%, figure 2, *p* < 0.001). The frequency dynamics of each genotype and the results for all experimental combinations are shown in electronic supplementary material, figure S2.

**Figure 3.**
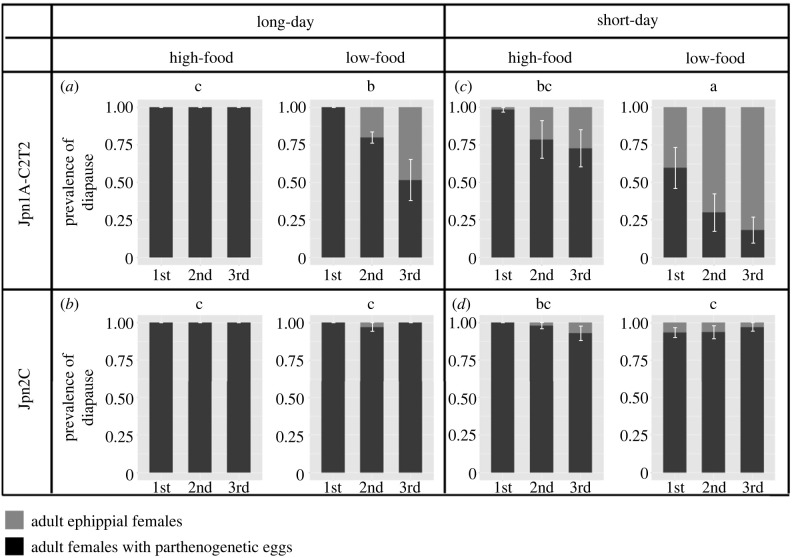
Prevalence of diapause, the ratio of ephippial females (grey) to the total number of reared individuals, at the first to the third clutches of each genotype under each photoperiodic condition: long-day (14 : 10 h light : dark) (*a,b*) and short-day (10 : 14 h light : dark) (*c,d*), and each food condition: high-food (0.63 mg C l^−1^) and low-food (0.063 mg C l^−1^). The prevalence of diapause was calculated based on the counts of ephippial females relative to ephippial females plus females with parthenogenetic eggs. The black and grey bars indicate adult females with clonal and diapausing eggs, respectively. The prevalence of diapause of each genotype in each condition was compared using the Tukey HSD test (a-b-c: *p* < 0.005). The error bars indicate the standard error.

In the life-history experiments, we confirmed that the shorter day length and low food availability induced the production of diapausing eggs (figure 3) as shown by previous studies [37–40]. Interestingly, the two genotypes of *D. pulex* showed different diapause induction responses to the photoperiodic conditions (figure 3). The Jpn1A-C2T2 genotype showed a higher prevalence of diapause in the short-day condition than the Jpn2C genotype, regardless of food concentration (figure 3*c*,*d*). The statistical analysis supported the effect of photoperiodic conditions on the prevalence of diapause (*p* < 0.001, table 1). The difference in diapausing response showed the effect of interaction between genotype and photoperiodic conditions (*p* = 0.013, table 1). In addition, the intrinsic growth rate was negatively correlated with the prevalence of diapause (*p* < 0.001. electronic supplementary material, figure S3a, table S1a). Similarly, the number of parthenogenetic offspring was negatively correlated with the number of ephippia from the first to the third clutch (*p* < 0.001, see figure S3*b* and table S1b in the electronic supplementary material). While the intrinsic population growth rate was not significantly associated with genotype and the interaction between prevalence of diapause and genotype, the number of parthenogenetic eggs was significantly associated with both genotype and the interaction between genotype and the number of ephippia (electronic supplementary material, figure S3b, table S1b).

**Table 1.** The difference in prevalence of diapause between genotypes, photoperiod and food concentration estimated by ANOVA (significance was considered at *p* < 0.05).

	d.f.	sum sq	mean sq	*F* value	Pr (>*F*)
genotype	1	1.553	1.553	40.065	<0.001
photoperiod	1	0.767	0.767	19.797	<0.001
food	1	1.017	1.017	26.238	<0.001
genotype : photoperiod	1	0.256	0.256	6.611	0.013
genotype : food	1	0.757	0.757	19.544	<0.001
photoperiod : food	1	0.026	0.026	0.674	0.416
genotype : photoperiod : food	1	0.017	0.017	0.439	0.511
residuals	48	1.860	0.039		

### (b) Mathematical analysis

**Figure 4.**
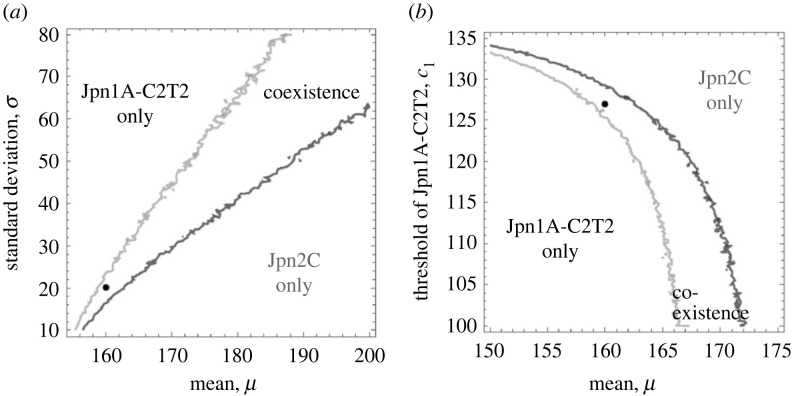
Effects of fluctuations in growing season length among years (*a*) and the diapause timing of the early-diapausing genotype Jpn1A-C2T2 (*b*) on coexistence of the two genotypes in the theoretical model. (*a*) Larger variation in the length of the growing season promotes stable coexistence of the two genotypes. (*b*) Earlier production of diapausing eggs of JPN1 promotes stable coexistence, whereas late production makes coexistence difficult. Horizontal axes are the mean of the normal distribution that the length of the growing season follows. Lines indicate parameter conditions where invasion growth rates (the logarithm of equation (2.4)) are zero. The invasion growth rate of Jpn1A-C2T2 is positive on the left of the black line, and that of Jpn2C is positive on the right of the grey line. Coexistence occurs in the area where the invasion growth rates of both Jpn1A-C2T2 and Jpn2C are positive (i.e. mutually invasible). The black points represent the parameter values in both figure 1*c* and 1*b*. The horizontal line in figure 4*b* indicates the parameter value in figures 1*c*,*b*, and 4*a*. We randomly sampled 10 000 parameter combinations, evaluated invasibility, and drew contour plots for the phase diagrams.

When we assume that the Jpn1A-C2T2 genotype switches to the diapausing phase earlier than the Jpn2C genotype based on the laboratory experiments (figure 3*a*,*b,* electronic supplementary material, appendix S1, figure S1), the two genotypes can coexist with the inter-annual fluctuations of the growing season length (figure 1*c*). Increasing the magnitude of inter-annual fluctuations of the growing season length (*σ*^2^) promotes coexistence of the two genotypes (figure 4*a*). Also, decreasing the parameter *c*_1_ expands the parameter space for coexistence because it results in an earlier production of diapausing eggs of the Jpn1A-C2T2 genotype and temporal niche differentiation with the Jpn2C genotype (with *c*_2_ = 0.75: figure 4*b*). On the other hand, increasing *b*_1_, the seasonal sensitivity of diapausing egg production of Jpn1A-C2T2 genotype can greatly expand the parameter space for coexistence (electronic supplementary material, figure S5a), while when decreasing *a*_1_, the maximum production of diapausing eggs of Jpn1A-C2T2 genotype slightly expands the parameter space for coexistence (electronic supplementary material, figure S5b).

## 4. Discussion

In the present study, we tested whether diapausing responses to photoperiodic conditions differ between two genotypes of *D. pulex* and whether these variations in photoperiodic responses can promote coexistence of the genotypes. Our results revealed intraspecific variation in diapausing responses to photoperiodic conditions and showed that this variation can contribute to the maintenance of genetic variation via the storage effect in the presence of inter-annual fluctuations of growth conditions, suggesting that the timing of diapause induction can be as significant as the timing of diapause termination. The inter-annual fluctuations in the length of the growing season can produce ‘good’ and ‘bad’ years for competing genotypes as the early-diapausing genotype is advantageous in ‘short’ years when winter comes earlier by assuring reproduction, whereas the late-diapausing genotype produces more diapausing eggs in ‘long’ years when winter comes later. It should be noted that the effects of ‘bad’ years may not be equivalent for the competing genotypes in the wild if the early-diapausing genotype produces fewer diapausing eggs in ‘long’ years whereas the late-diapausing genotype produces no or much less diapausing eggs in ‘short’ years. The genotype-specific responses to environmental fluctuations, together with overlapping generations due to dormancy, promote stable coexistence of genotypes via the temporal storage effect.

We found a trade-off between entering diapause and the intrinsic growth rate via clonal reproduction. Both intrinsic growth rate and the number of parthenogenetic offspring showed a negative correlation with the prevalence of diapausing and the number of ephippia, respectively (electronic supplementary material figure S3, table S1). This result suggests that the higher prevalence of diapausing of the Jpn1A-C2T2 genotype (figure 3) decreases its asexual population growth rate, thereby making the Jpn2C genotype dominant in the clonal reproduction experiment (figure 2). Since the number of parthenogenetic offspring was significantly associated not only with genotype but also with the interaction between genotype and the number of ephippia (electronic supplementary material figure S3b, table S1b), the trade-off between parthenogenetic reproduction and diapause possibly differs depending on genotypes. The observed negative association between the number of ephippia and parthenogenetic female offspring as well as the induced investment on ephippia by photoperiods as a cue of unfavourable environments are consistent with recent experimental studies [38,57].

The simulation analysis supported the idea that the variation in timing of diapause induction promotes coexistence. It shows that the coexistence region increases because the Jpn1A-C2T2 genotype induces diapause relatively earlier than the Jpn2C genotype (figure 4*b*). As this early induction of diapause may be advantageous in years with short growth periods (when winter comes earlier than usual years: figure 1), inter-annual fluctuations of the growth period can promote the maintenance of genetic variation through temporal niche partitioning (figure 4) [2,33]. Therefore, our study suggests that photoperiodic conditions may be one of the mechanisms that differentiate phenology [58]. Furthermore, the present study suggests that not only the timing of diapause termination [17,20–22,59] but also the timing of diapause induction is important to maintain coexistence via the storage effect.

Previous studies have investigated adaptive evolution of the optimal timing of diapause induction by using theoretical and empirical approaches (e.g. [23,24]). Particularly, Hairston and Dillon intensively studied timing of diapause induction in copepods (*Diaptomus sanguineus*). They found that diapause is induced by photoperiod [60] one generation prior to catastrophic events (e.g. the initiation of seasonally intense fish predation) to optimize their fitness [24]. Thus, the observed different timing of diapause induction in our study may also be due to local adaptation to different timing of catastrophic events in different lakes from which each genotype migrated to Lake Fukami-ike [61]. Furthermore, when the catastrophic timing varies from year to year, they found that a bet-hedging strategy may be better than an environmentally cued strategy ([25], see also [26]). Therefore, while our study proposed that genetic variation is stably maintained due to temporal fluctuations in the length of growing season, it may not be evolutionarily stable: after a long period of time, two genotypes may eventually employ the bet-hedging strategy over environmentally-induced diapause and stable coexistence may be lost. It will be interesting to consider how short-term and long-term evolution interact with coexistence of competing genotypes and species in future studies [62,63].

While our study showed that the early-diapausing genotype decreases production of clonal offspring due to the trade-off between diapause and clonal reproduction, the opposite situation (i.e. the growth rate of clonal reproduction of an early-diapausing genotype is higher than that of a later-diapausing genotype) may also promote stable coexistence. This works when the genotype with faster clonal reproduction shows density-dependent diapause induction [64]. As diapausing individuals do not reproduce, the density-dependent production of diapausing stages results in a negative density-dependent population growth (i.e. a population with a high density induces diapause and stops growing). This negative density-dependent population growth of the early-diapausing genotype can promote coexistence, as the late-diapausing genotype can grow without competition after the diapause induction of the early-diapausing genotype (‘protection effect’ [64,65]). Therefore, inter-annual environmental variation and the storage effect are not necessary for the coexistence in this case. It would be interesting to compare the two coexistence mechanisms (i.e. the storage effect and density-dependent diapause) in future studies.

There are other coexistence-promoting factors that we did not evaluate in this study. First, variation in reproductive success and the hatching timing can affect the storage effect [17–19]. Although we implicitly assumed that the hatching from the diapausing egg bank occurs simultaneously in the two genotypes in our model, earlier hatching of the Jpn1A-C2T2 might further promote coexistence by allowing a temporal niche differentiation with Jpn2C. Photoperiodic conditions also affect timing of diapause termination ([66] but see [67–69]). Furthermore, recent studies demonstrated that population growth rates of different *Daphnia* lineages may vary seasonally in response to temperature and food quality [36], and thus the inter-annual variation of reproductive success may also be an important factor. Second, we did not measure the minimum resource requirement for population growth, R* [70], lineage-specific mortality rates and functional responses [71]. Thus, while we primarily focused on the temporal storage effect in this study, it may be possible for relative nonlinearity to promote coexistence [72–74]. Furthermore, different responses of lineages to predators and primary producers possibly affect coexistence. Two lineages, JPN1 and JPN2, may have different defence traits against predation (e.g. *Chaoborus*) [75], and different population growth rates when feeding on blue-green algae, cyanobacteria [36] (note that we fed green algae, *Scenedesmus*, in the laboratory experiments). Although we evaluated the clonal reproduction of two genotypes by short-term experiments in this study, long-term experiments or field observation is necessary to examine the outcome of competition (e.g. [76]). Further long-term (multigenerational) experiments and field observations may enable us to empirically test the relative contributions of different coexistence mechanisms including the resource differentiation, lineage-specific responses to predators, the storage effect and relative nonlinearity [74–79].

In addition, unfortunately, we could not observe the ongoing population dynamics of the two genotypes in Lake Fukami-ike and we cannot determine whether the two genotypes induce diapause with different timing in the wild. The population of *D. pulex* has reduced drastically (or gone extinct) in Lake Fukami-ike in recent years [44], probably due to the increase of planktivorous invasive fish (*Lepomis macrochirus*). Moreover, in Lake Fukami-ike, the period when two *D. pulex* genotypes cooccurred was relatively short, and it is not possible to examine whether the intraspecific difference of diapause induction could promote long-term coexistence. It will be fruitful to observe the dynamics of active populations and egg banks and conduct long-term field studies in other lakes where multiple competitive genotypes coexist in future studies. Researchers should be aware that, while photoperiodic stimuli are in general credible signals of seasonal changes [54], recent climate change may affect the relationship between diapause induction and competitive interactions, as well as the relationship between the hatching of diapausing eggs and competitive interaction [80]. Rudolf [58] suggested that shifts in phenology can affect species interaction, including coexistence, and responses to climate change. Thus, stable coexistence of two genotypes may become difficult in future environments under the ongoing climate change.

As discussed above, there are many traits to be measured in future studies, but our mathematical framework will be helpful to understanding how various trait combinations and the resultant trade-offs affect coexistence. By extending the model to incorporate spatial dynamics, it may be possible to examine whether the intraspecific difference in diapausing strategy can affect migration and range expansion. In Lake Fukami-ike, paleolimnological and population genetic analyses of diapausing eggs revealed that Jpn2C was established earlier than Jpn1A-C2T2 [45]. Further evolutionary and genetic research can contribute to our understanding of evolutionary dynamics with intraspecific interaction and spatial competition–colonization trade-off [81]. Furthermore, comparing the diapause response between a lake with multiple lineages and a lake with a single lineage will be effective to examine the adaptive response to intra-specific competition (i.e. intra-specific ecological character displacement: [19]). In addition, long-term in-depth analysis of field data is necessary to test our hypothesis: whether different timing of diapause induction based on photoperiodic responses can promote stable coexistence of multiple genotypes. Monitoring frequency dynamics of diapausing eggs of each genotype in addition to active populations throughout a year will be one of the best approaches for understanding coexistence mechanisms.

## 5. Conclusion

This study showed that two genotypes of *D. pulex* in Japan have different diapausing responses to photoperiodic conditions. Although stopping clonal reproduction earlier by producing diapausing eggs might decrease total clonal production, this variation in diapausing strategy can promote coexistence of the genotypes through the temporal storage effect. Our results suggest that the timing of diapause induction can be as significant as the timing of diapause termination for the storage effect and contribute to deepening our understanding of how genetic and species diversity are maintained in natural environments.

## Data Availability

The datasets are available from Dryad Digital Repository: https://doi.org/10.5061/dryad.69p8cz977 [82]. Supplementary material is available online [83].
